# Integrative Analysis of Membrane Proteome and MicroRNA Reveals Novel Lung Cancer Metastasis Biomarkers

**DOI:** 10.3389/fgene.2020.01023

**Published:** 2020-08-28

**Authors:** Yan Kong, Zhi Qiao, Yongyong Ren, Georgi Z. Genchev, Maolin Ge, Hua Xiao, Hongyu Zhao, Hui Lu

**Affiliations:** ^1^SJTU-Yale Joint Center for Biostatistics and Data Science, Department of Bioinformatics and Biostatistics, School of Life Sciences and Biotechnology, Shanghai Jiao Tong University, Shanghai, China; ^2^State Key Laboratory of Microbial Metabolism, Joint International Research Laboratory of Metabolic and Developmental Sciences, School of Life Sciences and Biotechnology, Shanghai Jiao Tong University, Shanghai, China; ^3^Center for Biomedical Informatics, Shanghai Engineering Research Center for Big Data in Pediatric Precision Medicine, Shanghai Children’s Hospital, Shanghai, China; ^4^Bulgarian Institute for Genomics and Precision Medicine, Sofia, Bulgaria; ^5^State Key Laboratory of Medical Genomics, Shanghai Institute of Hematology, Rui Jin Hospital, School of Medicine and School of Life Sciences and Biotechnology, Shanghai Jiao Tong University, Shanghai, China; ^6^Department of Biostatistics, Yale University, New Haven, CT, United States

**Keywords:** membrane proteome, lung cancer metastasis, multi-omics analysis, microRNA, prognostic

## Abstract

Lung cancer is one of the most common human cancers both in incidence and mortality, with prognosis particularly poor in metastatic cases. Metastasis in lung cancer is a multifarious process driven by a complex regulatory landscape involving many mechanisms, genes, and proteins. Membrane proteins play a crucial role in the metastatic journey both inside tumor cells and the extra-cellular matrix and are a viable area of research focus with the potential to uncover biomarkers and drug targets. In this work we performed membrane proteome analysis of highly and poorly metastatic lung cells which integrated genomic, proteomic, and transcriptional data. A total of 1,762 membrane proteins were identified, and within this set, there were 163 proteins with significant changes between the two cell lines. We applied the Tied Diffusion through Interacting Events method to integrate the differentially expressed disease-related microRNAs and functionally dys-regulated membrane protein information to further explore the role of key membrane proteins and microRNAs in multi-omics context. *Has-miR-137* was revealed as a key gene involved in the activity of membrane proteins by targeting MET and PXN, affecting membrane proteins through protein–protein interaction mechanism. Furthermore, we found that the membrane proteins CDH2, EGFR, ITGA3, ITGA5, ITGB1, and CALR may have significant effect on cancer prognosis and outcomes, which were further validated *in vitro*. Our study provides multi-omics-based network method of integrating microRNAs and membrane proteome information, and uncovers a differential molecular signatures of highly and poorly metastatic lung cancer cells; these molecules may serve as potential targets for giant-cell lung metastasis treatment and prognosis.

## Introduction

Lung cancer remains the leading cause of cancer-related mortality in the world, with an overall 5-year survival rate of 18% (Siegel et al., 2017). In 2016, over 155,000 people died from lung cancer in the United States alone (Siegel et al., 2017). These low survival rates are partly due to the fact that over 50% of patients are diagnosed at a later stage, for which the 5-year survival is only 4%. Approximately 80–85% of lung cancer cases are non-small cell lung cancer (NSCLC), and the remaining 15–20% are small-cell lung cancer cases (Giaccone and Zucali, 2008). In NSCLC, it is estimated that over 40% of patients have metastases at the time of diagnosis (Waqar et al., 2018). The prognosis is poor in metastatic cases -only ∼1% of such NSCLC patients will survive five or more years (Borghaei et al., 2017).

Cancer metastasis involves tumor cell invasion across interstitial tissues and basement membranes (Jiang et al., 2015; Qiao et al., 2019). Abnormal expression of membrane proteins in cancer tissues and cells has been shown to play a key role in cancer occurrence and metastasis (Lethlarsen et al., 2009; Kampen, 2011). In a recent study of hepatocellular carcinoma (HCC), golgi membrane protein 1 (GOLM1) was shown as a key target of miR-382, HCC cells metastasis status was inhibited when GOLM1 is down-regulated in HCC cells (Zhang et al., 2018). *N*-cadherin (CDH2), a direct target of miR-145, is a cell-cell adhesion molecule that contributes to the invasive/metastatic phenotype in many cancers such as gastric cancer, breast cancer, and lung cancer (Lei et al., 2017; Mo et al., 2017; Ye et al., 2018). β-catenin (CTNNB1), involved in the regulation of cell adhesion, promote ovarian cancer metastasis and liver cancer (Arend et al., 2013; Ding et al., 2014). Mucin 1(MUC1) protein, a membrane-tethered mucin glycoprotein, is also associated with poor prognosis and enhanced metastasis in human pancreatic cancers (Wu et al., 2018). In addition, microRNAs have important roles in cancer metastasis (Nicoloso et al., 2009), and multiple microRNAs, such as hsa-miR-1, hsa-miR-217, hsa-miR-206, and has-miR-577 were previously shown to play key roles in cancer metastasis (Liu et al., 2015; Chen et al., 2017; Samaeekia et al., 2017).

The complex regulatory landscape of cancer metastasis underscores the need of integrative approaches in cancer research. Multi-omics computational studies are an active area of investigation and perform analysis of genomic, proteomic, and transcriptional data combined with prior knowledge of regulatory relationships to uncover clinically relevant discovery such as biomarkers, therapeutically targets, and outcome prediction (Liu et al., 2017; Jiang et al., 2019; Xu et al., 2020). One such recently proposed method – the Tied Diffusion Through Interacting Events (TieDIE) algorithm uses a network diffusion approach to connect genomic perturbations to transcriptional changes (Paull et al., 2013). With the help of the TieDIE algorithm, a contributing factor (small GTPase RHEB) to the differences observed between BRAF and RAS mutants was discovered; in another cancer study, researchers combined transcriptional regulators, mutated genes, and differentially expressed kinases with TieDIE and synthesized a robust signaling network which consists of drug-able kinase pathways (Drake et al., 2016).

Although both membrane proteome and microRNA have been shown of great importance previously, there was no systematic combined analysis using membrane proteome together with microRNA data on lung squamous cell carcinoma (LUSC). In this study, we utilized quantitative membrane proteome and microRNA expression together with multiple regulation networks to perform comparative analysis of highly and poorly metastatic lung cancer cell lines (95C and 95D). In the following, we described the methodology used for experiment and computational analysis; differentially expressed membrane proteins are identified, then using joint analysis method to integrate microRNA expression data. Finally the significance of the study is discussed.

## Materials and Methods

**FIGURE 1:**
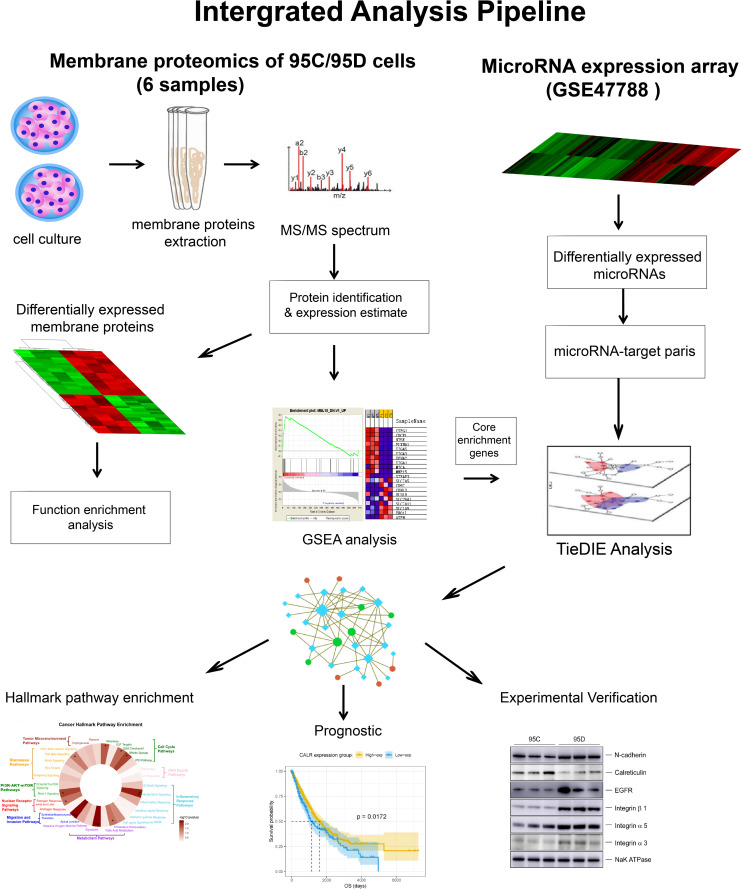
Overall workflow of multi-omics integrative analysis.

The methods utilized in the work aim to discover biomarkers which are associated with disease outcomes measured by overall survival (OS). We integrated experimental and computational approaches, proteomics and genomics (microRNA) data, and bioinformatics analysis to drive bio-medical discovery. The overall work-flow is depicted in Figure 1.

### Experimental Data Collection – Protein Identification and Quantification

#### Cell Culture and Membrane Protein Preparation

Human giant cell lung cancer cell lines of poorly (95C) and highly (95D) metastatic potential were purchased from the Institute of Biochemistry and Cell Biology of the Chinese Academy of Sciences (Shanghai, China). Cells were cultured in DMEM and RPMI 1640 medium supplemented with 10% fetal bovine serum for 95D and 95C cells respectively at 37°C and 5% CO_2_ incubator. All culture media were supplemented with 100 U/mL penicillin and 100 mg/mL streptomycin sulfate. All cultured cells were tested for mycoplasma contamination before use. Membrane proteins isolation was performed with the Pierce^®^ Cell Surface Protein Isolation Kit (Pierce, Thermo Fisher Scientific, United States), and followed the protocol described by de Wit et al. (2012). All reagents were cooled to 4°C before protein biotinylation. The cells were washed twice with ice-cold phosphate buffered saline (PBS) followed by incubation with 0.25 mg/mL Sulfo-NHS-SS-biotin (Pierce) in 48 mL ice-cold PBS per flask on orbital shaker for 30 min at 4°C. Then, 500 μL of quenching solution were added to each flask to quench the reaction. After being washed with ice-cold PBS, harvested by gentle scraping, and pelleted by centrifugation, the cells were lysed using the Lysis Buffer (Pierce) which was added with protease inhibitors for 30 min on ice with vortexing every 5 min for 5 s. The cell lysates were centrifuged at 10,000 × *g* for 2 min at 4°C to remove cell remnants. Before clarified supernatant was used to purify biotinylated proteins on NeutrAvidin Agarose (Pierce), 500 μL of NeutrAvidin Agarose slurry was added and centrifuged 1 min at 1,000 × *g* and the flow-through was discarded followed by washing with Pierce Wash Buffer in a provided column (Pierce) trice. The clarified supernatant was added and incubated for 2 h at 4°C using an end-over-end tumbler to mix vigorously and allow the biotinylated proteins to bind to the NeutrAvidin Agarose slurry. Unbound proteins were removed by washing with 1% Non-idet-P40 and 0.1% SDS in 500 μL PBS thrice and then by washing with 0.1% Non-idet-P40 and 0.5 M NaCl in 500 μL PBS trice. Finally, the captured proteins were eluted from the biotin-NeutrAvidin Agarose and were collected by column centrifugation at 1,000 × *g* for 2 min. Three biological replicates were obtained for both cell lines. All protein concentrations were quantified using the BCA protein Assay Kit (Pierce) and the lysates were stored at −20°C for further analysis.

#### Liquid Chromatography Tandem Mass Spectrometry (LC-MS/MS)

Equal amount of proteins was digested overnight at 37°C by trypsin (Promega, Madison, WI, United States) using the FASP approach. Briefly, 30 μg membrane proteins were used and three biological repeats of each cell line were prepared. Equal amount of peptide was injected into Easy-nLC 1,000 m (Thermo Scientific) coupled with a Q-Exactive mass spectrometer (Thermo Fisher Scientific) (Michalski et al., 2011; Kelstrup et al., 2012). Peptides were eluted to analytical column (75 μm × 15 cm) packed with Jupiter Proteo resin (3 μm, C18, 300 Å, Phenomenex, Torrance, CA, United States). The mobile phase consisted of buffer A (2% acetonitrile and 0.1% formic acid in water) and buffer B (0.1% formic acid in 95% acetonitrile). A flow rate of 250 nL/min and 60 min of the gradient from 12% B to 32% B was applied for the separation of peptides. MS Scan range was from 300 to 1,600 m/z with the resolution of 70,000. For MS/MS, scan range was from 200 to 2,000 m/z with the resolution of 17,500. We performed full MS scan in a positive mode and then selected the five most dominant icons from the initial MS scan for collision-induced dissociation.

#### Protein Identification and Quantitation

To identify proteins from the acquired data, MS/MS spectra were searched against the Human SwissProt database (548, 208 sequences) (Jungo et al., 2012) using the MASCOT software (version 2.0) (Matrix Science, London, United Kingdom). SwissProt database is a high quality manually annotated and non-redundant protein sequence database, and now more and more bioinformatics data mining algorithms are designed using this database (Le et al., 2017a,2019a).

The parameters for searching were the MASCOT defaults – enzyme of trypsin, two missed cleavage, fixed modifications of carbamidomethyl (C), and variable modifications of oxidation (M). We set mass tolerance to 20 ppm for MS precursors and 0.05 Da for fragment ions, and then peptide charges of +2, +3, and +4 were retained. For protein identification, we used *p*-value less than 0.05 and false discovery rate (FDR) less than 0.01 at the protein level as the criteria to distinguish two peptides. Label-free quantification was performed by intensity-based absolute quantification (iBAQ), which was based on at least two unique peptides to quantify the different protein profiling in the 95C cell and 95D cell membrane. Quantile normalization was performed to ensure that each sample had the same distribution, the two-fold change and *p*-value less than 0.01 cut-off was set up for the screening of differentially expressed proteins.

### Bioinformatics Analysis

#### Protein Subcellular Localization Annotation and Transmembrane Domain Prediction

The first step of the bioinformatics analysis aimed to identify membrane proteins. Here, we used the following process to annotate the membrane proteins. First, Gene Ontology (GO) cellular component annotation of all identified proteins was performed by the R *go.db* package. The GO is a human and machine readable gene annotation resource, which has been widely used to enable computational discovery in diverse areas such as protein function identification (Le et al., 2017b,2019b), text mining in life sciences (Przybyla et al., 2016), and underlying molecular disease mechanisms (Kramarz et al., 2020). Second, additional subcellular location information was downloaded from the UniProt database (Jungo et al., 2012) and added to the protein subcellular localization annotation. Third, transmembrane domains in all identified membrane proteins were predicted by TMHMM^1^ Serve v.2.0 (Krogh et al., 2001). This gave us a list of 3,240 membrane proteins which were utilized in the downstream analysis.

#### Differential Expression Analysis and Enrichment Analysis

In the second step of the bioinformatics analysis we searched for protein differential expression and enrichment. Utilizing the Student’s *t*-test, we tested the 3,240 membrane proteins for differential expression between poorly metastatic 95C and highly metastatic 95D cell lines. Proteins with a *p*-value <0.05 and |log_2_FC| > 1 were considered to be significantly differentially expressed and included in the reduced set (*n* = 163) for further analysis. GO and KEGG pathway enrichment analysis by the *clusterprofiler* R package (Yu et al., 2012) was performed on the differentially expressed membrane proteins in order to understand which function they may affect. Gene set enrichment analysis (GSEA) were performed by the GSEA software v.3.0 (Subramanian et al., 2005) using the molecular signatures database MSigDB (Liberzon et al., 2011). At the end of this step we obtained a list of 163 differentially expressed proteins and 87 enriched genes.

#### Multi-Omics Data – MicroRNA

To integrate genomics data into our analysis, microRNA expression array data of 95D and 95C cells (GSE47788) was downloaded from the Gene Expression Omnibus (GEO) database (Edgar et al., 2002; Wang X. M. et al., 2013). We directly used the differential expression results provided by the study. For the 64 differentially expressed microRNAs, we applied the miRWALK2.0 (Dweep and Gretz, 2015) software to build a list of microRNAs and their gene targets.

#### Generation of Biological Pathway Subnetwork Connecting MicroRNA and Enriched Genes

Having obtained (1) the set of 87 enriched genes and (2) the set 64 of differentially expressed microRNA and their gene targets, we build a sub-network which significantly close-connects these genes and microRNAs. We used the *Tied Diffusion through Interacting Events* (TieDIE) software (Paull et al., 2013; Drake et al., 2016). The Multinet pathway database (Brown et al., 2013) together with the validated microRNA-target pairs selected by miRWALK2.0 in the previous step served as the background network of the TieDIE program. 32 membrane protein genes and 180 linker genes were selected in this step.

#### Cancer Hallmarks Enrichment Calculation

The set of genes in the sub-network built with TieDIE in the previous step were used to perform cancer hallmark enrichment which is now a popular functional analysis method for a gene clustering (Drake et al., 2016; Ge et al., 2020). Cancer hallmark definitions were also downloaded from the MSigDB database, and the cancer hallmark pathway enrichment were performed by calculating the probability of overlap between input genes and the hallmark pathway gene sets and evaluated by hyper geometric test with Benjamini and Hochberg (1997) correction of p-values. The source code for cancer hallmark enrichment analysis is publicly available at: https://github.com/YankongSJTU/CHEA.

#### Survival Analysis

Integrating the above-obtained gene lists with survival data, we performed patient survival analysis to determine if the selected genes have impact on cancer related outcomes. We downloaded mRNA expression data (FPKM values) for the 32 membrane protein genes from the Human Protein Atlas^2^ (Uhlen et al., 2010) of lung adenocarcinoma (LAC) and LUSC patients (Tomczak et al., 2015), 925 samples in total. From the same source we also obtained patient OS data. For each gene, FPKM values from the 20th to 80th percentiles were used to group the patients; significant differences in the survival outcomes of the groups were examined and the value yielding the lowest log-rank *p*-value was set to be the best cut-off value. Patients were classified into two groups: group 1 with values above the cutoff (high expression level group) and group 2 with values below the cutoff (low expression level group). The outcome differences for each group were calculated using the Log-rank test by the Kaplan–Meier method (Bland and Altman, 1998). Prognostic analysis was performed by using the R packages *KMsurv*, *survival*, and *survminer* (Latouche and Aurelien, 2019). All p-values were derived from two-tailed statistical tests, and *p*-value <0.05 was considered as statistically significant. At the completion of the bioinformatics analysis, six significant gene biomarkers significantly correlated with OS were determined.

We also constructed a prognostic risk index with these selected membrane proteins:

(1) $$\mathrm{Risk}\,\mathrm{Index}=\sum \text{expression}\,\left({\text{P}}_{\text{i}}\right)*\frac{{\text{HR}}_{\text{i}}}{\text{maximum}\,\left(\text{expression}\,\left({\text{P}}_{\text{i}}\right)\right)}$$

where P_*i*_ (i = 1,2,3,…,K) means the selected K membrane proteins. Samples are grouped according to the risk factor levels, and the prognosis differences are compared.

### Experimental Verification

The cellular expression of the six candidate proteins were verified experimentally via Western Blot analysis. Total cell lysates were obtained using RIPA buffer (Thermo Fisher Scientific, United States). Proteins were separated by SDS-polyacrylamide gel electrophoresis (SDS–PAGE) and transferred to polyvinylidene fluoride (PVDF) membrane (Millipore, Burlington, MA, United States). The membranes were blocked in PBS, 10% (w/v) skim milk for 1 h in phosphate buffer saline-Tween 20 (PBS-T), and incubated for 3 h at RT in 5% milk in PBS-T with primary antibodies: CDH2, EGFR, ITGA3, ITGA5, ITGB1, and CALR (Abcam, Cambridge, United Kingdom). Then, after washing, the PVDF membrane was incubated with secondary antibody (The Jackson Laboratory, United States) for 40 min at RT. Thermo Scientific SuperSignal West Pico PLUS Chemiluminescent Substrate kit (Thermo Fisher Scientific, United States) was used for visualization.

## Results and Discussion

### Protein Identification and Differential Expression Analysis

We first sought to determine the full set of membrane proteins detected by mass spectrometry and then identify the subset of differentially expressed ones. A total of 3,241 unique proteins were identified and quantified. A total of 3,107 proteins (95.9%) were annotated by GO cellular component analysis and 2,887 proteins (89.1%) were annotated by using the UniProt subcellular localization database. Subcellular localization annotation analysis predicted that 1,762 proteins were membrane proteins (54.7%), whereas the TMHMM algorithm predicted that 590 proteins (18.3%) had a transmembrane domain. Significant differences between 95D and 95C cells were observed in the expression of 163 membrane proteins (|log_2_FC| > 1 and *p*-value < 0.05). We provide detailed subcellular localization annotations together with TMHMM results of all proteins in Supplementary Table S1. All differential expression results are listed in Supplementary Table S2.

### Functional Characterization of Differentially Expressed Membrane Proteins

Differential expressed membrane proteins may play a key role in tumor metastasis. Membrane proteins as well as extra-cellular matrix (ECM) molecules, cell adhesion molecules and adhesion receptors form into functional complex units and maintain cell–cell adhesions. These complexes, once disassembled, will increase tumor metastasis and invasion (Gumbiner, 1996; Vadakekolathu et al., 2018). To further examine the mechanistic role in cancer metastasis of differentially expressed membrane proteins, we performed two-step functional enrichment analysis.

First, GO and KEGG functional enrichment analysis revealed that differentially expressed membrane proteins were mainly centered on focal adhesion and cell-substrate adherens junctions, and many metabolic pathways. For example, the three most significantly enriched GO terms were GO:0005925∼focal adhesion (*q*-value = 7.2e−43), GO:0005924∼cell-substrate adherens junction (*q*-value = 7.2e−43), and cell-substrate junction (*q*-value = 1.2e−42), and the three most significantly enriched KEGG pathway terms were hs03010∼Ribosome (*q*-value = 1.07e−16), hsa05412∼Arrhythmogenic right ventricular cardiomyopathy (ARVC) (*q*-value = 1.65e−4), and hsa05416∼Viral myocarditis (*q*-value = 3.22e−3). All detailed enrichment results are shown in Supplementary Table S4.

In the second step of this analysis, GSEA revealed that differentially expressed membrane proteins were enriched in 13 GO categories (MSigDB c6 Gene Ontology or GO categories) including of GO_POSITIVE_REGULATION_OF_LOCOMOTION and GO_CELL_JUNCTION_ORGANIZATION. KEGG_FOCAL_ADHESION pathway was also enriched. Similar molecular terms, “KEGG_FOCAL_ ADHESION” and “REACTOME_HEMOSTASIS” were also identified using other functional gene sets (MSigDB c6 KEGG or Reactome categories) (Table 1). Of all 87 enriched genes, 21 were considered as a core enrichment set, i.e., genes which were considered to contribute the most to the enrichment result According to the differential expression analysis, all core enrichment genes were significantly up-regulated, including ALCAM, LDOA, TP1A1, TP1B1, TP1B3, CDH2, CTNNB1, CXADR, EGFR, EPHA2, GLG1, ITGA3, ITGA5, ITGB1, ITGB3, JAM3, MPZL1, NEGR1, PARK7, PTPRF, and SLC16A3 (see Supplementary Table 5).

**TABLE 1:** Gene set enrichment result of differentially expressed membrane proteins.

GS(follow, link, to, MSigDB)	Size	ES	NES	NOM, *p*-value	FDR, *q*-value
GO_POSITIVE_REGULATION_OF_DEVELOPMENTAL_PROCESS	17	0.49060193	2.2359705	0	0.044999983
GO_POSITIVE_REGULATION_OF_LOCOMOTION	15	0.52440554	2.2129607	0	0.044999983
GO_CELL_PROJECTION_PART	18	0.46777532	2.201335	0	0.044999994
GO_PLASMA_MEMBRANE_REGION	24	0.5549008	2.1945791	0	0.044999983
GO_CELLULAR_COMPONENT_MORPHOGENESIS	16	0.51833624	2.1498182	0	0.044999994
KEGG_FOCAL_ADHESION	17	0.45756185	1.9556208	0	0.044999994
REACTOME_HEMOSTASIS	26	0.38783315	1.9427094	0	0.045000017
GO_CELL_JUNCTION_ORGANIZATION	16	0.48022297	1.9331897	0	0.044999983
GO_REGULATION_OF_CELL_DIFFERENTIATION	18	0.49768242	1.9189458	0	0.044999994
GO_MEMBRANE_MICRODOMAIN	15	0.5177578	1.9009451	0	0.044999994
GO_TISSUE_DEVELOPMENT	32	0.38096464	1.8797828	0	0.045
GO_ANATOMICAL_STRUCTURE_FORMATION_INVOLVED_IN_MORPHOGENESIS	18	0.3993144	1.8518116	0	0.044999994
GO_REGULATION_OF_PHOSPHORUS_METABOLIC_PROCESS	23	0.42798108	1.8506685	0	0.045000024
GO_REGULATION_OF_PROTEIN_MODIFICATION_PROCESS	21	0.46240425	1.8467267	0	0.045000017
GO_ENZYME_LINKED_RECEPTOR_PROTEIN_SIGNALING_PATHWAY	15	0.5241594	1.8411065	0	0.04499999
GO_RECEPTOR_BINDING	24	0.39534718	1.8257784	0	0.044999983
GO_MEMBRANE_REGION	31	0.420572	1.8051884	0	0.051817805
GO_PROTEIN_COMPLEX_BINDING	28	0.36254478	1.787877	0	0.051439002
GO_REGULATION_OF_CELLULAR_COMPONENT_MOVEMENT	24	0.39252102	1.7859758	0	0.05110011

*ES, NES, and FDR are enrichment score, normalized ES, and false discovery rate, respectively.*

### MicroRNAs and Significant Sub-Network Derived From TieDIE Program

MicroRNAs are non-coding RNAs which participate in cellular activity by regulating target genes. Increasing number of studies have reported that microRNAs are frequently differentially expressed in numerous types of human cancer and play an important role in the progression and development of NSCLC (Inamura and Ishikawa, 2016; Berrout et al., 2017; Chang et al., 2017; Zhang et al., 2017; Iqbal et al., 2018). Although microRNAs act only in the cell where they are synthesized, they can also influence the functions of neighboring cells or play a role in the tumor micro-environment by modulating the ECM state (Rutnam et al., 2013).

Utilizing the TieDIE program (a pathway-based multi-omics method which extends on the heat diffusion strategies and uses a network diffusion approach to connect proteins and genes related to diseases) we were able to link differentially expressed microRNAs with membrane proteins.

**FIGURE 2:**
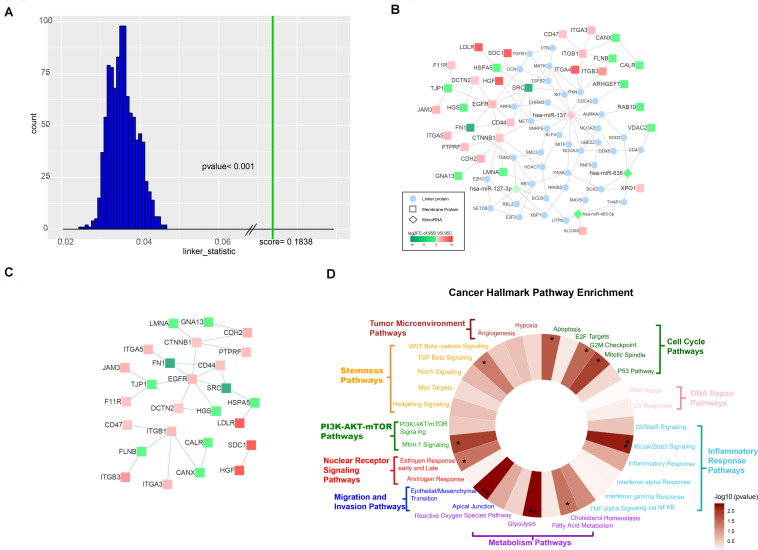
Characterization of the TieDIE network. **(A)** The distribution of background scores and real score. The distribution of background scores is shown as blue bars, while the green line represents the real score. **(B)** Compact subnetwork after manually deleting linker proteins whose degree was one from the raw subnetwork constructed by the TieDIE program. The rhombus nodes represent for microRNAs, the circle nodes represent for linker proteins, while the square nodes represent differentially expressed membrane proteins. The node color changes according to the fold change values. **(C)** PPI subnetwork of 32 differentially expressed membrane proteins. **(D)** Wheel plot of cancer hallmark enrichment of the TieDIE subnetwork.

First, 64 differentially expressed microRNAs were obtained directly from a previous study (Supplementary Table S2). We successfully found 300 targets of 64 microRNAs with the help of MIRWALK 2.0 and synthesized a validated microRNA-regulated target network (Supplementary Table S3) which served as background database together with the Multinet database (Khurana et al., 2013). The input membrane proteins and microRNAs were found to be significantly close (*p* < 0.001) in a pathway space based on a background model generated by 1,000 permutations of the data (Paull et al., 2013), where each input set (membrane proteins, microRNAs) was swapped with genes of similar network connectivity while the other one was fixed (Figure 2A).

We selected a compact sub-network with a high level of specificity, which consisted of 216 nodes – four microRNAs (hsa-miR-137, hsa-miR-483-5p, hsa-miR-638, and hsa-miR-127-3p), 32 differentially expressed membrane proteins (HSPA5, CANX, TJP1, FN1, ITGA3, ITGA4, ITGA5, XPO1, JAM3, F11R, SDC1, FLNB, DCTN2, VDAC2, ALCAM, RAB10, SRC, LDLR, CDH2, EGFR, CTNNB1, ITGB1, ITGB3, CD44, CD47, HGS, ARHGEF7, HGF, LMNA, CALR, PTPRF, and GNA13) and 180 linking proteins connected by 244 edges (Supplementary Table S6). We manually deleted those linker proteins whose degree was 1 and constructed a sub-network consisting of four microRNAs, 32 membrane proteins, and 38 linker proteins with 101 edges (Figure 2B). Focusing on differentially expressed membrane proteins, we found 32 membrane proteins which were connected through protein–protein interactions (PPI) directly (Figure 2C). Regarding the four microRNAs in the sub-network (hsa-miR-137, hsa-miR-483-5p, hsa-miR-638, and hsa-miR-127-3p) – only hsa-miR-137 was up-regulated in the 95D cell line and the other three were down-regulated in the 95D cell lines as comparing with the 95C cell lines.

We also found that the four sub-network microRNAs were not independent of each other but connected by at least one linker protein. The three down-regulated microRNAs were previously reported to be associated with tumor metastasis in recent studies, and to influence the expression of both XPO1 and ALCAM (Ma et al., 2014; Herr et al., 2017; Shi et al., 2018; Yue et al., 2018). We focused on has-miR-137, which was up-regulated in the high-metastatic (95D) cell line and increases invasion and metastasis of NSCLC cells (Chang et al., 2017) Four up-regulated membrane proteins, HGF, CTNNB1, ITGB1, and ITGA4, involved in focal adhesion pathway, were linked to has-miR-137 by two mediation genes – PXN and MET (Figure 2B). Paxillin (PXN), whose expression was negatively correlated with has-miR-137 (Jiang et al., 2018), encodes a focal adhesion-associated protein and plays an important role in signal transduction, regulation of migration, proliferation and apoptosis. MET encodes tyrosine-protein kinase Met (c-Met) which possesses tyrosine kinase activity and is a well-characterized driver of oncogenesis occurs in multiple cancers include of NSCLC (Gentile et al., 2008). In this study, we found that MET and PXN, which are regulated by has-miR-137, may affect membrane proteins through PPI. Although proto-oncogene tyrosine-protein kinase SRC was found to be down regulated in the high-metastasis (95D) cell line in this study, many other research indicated SRC was highly expressed in NSCLC (Giaccone and Zucali, 2008; Rothschild et al., 2010).

### Cancer Hallmark Pathway Enrichment

We show that our TieDIE sub-network is significantly close in the pathway space. Considering the cancer hallmark pathway enrichment of all proteins involved in the TieDIE sub-network (32 differentially expressed membrane proteins and 180 linker proteins), we found the five main cancer hallmark pathway categories were significantly enriched, including of cell cycle pathway category (*p*-value = 0.0154), inflammatory response pathway category (*p*-value = 0.0105), metabolism pathway category (*p*-value = 0.0196), migration and invasion pathway category (*p*-value = 5.8380e−06), and PI3K/AKT mTOR pathway category (*p*-value = 0.0292) (Table 2).

**TABLE 2:** Cancer hallmark pathway enrichment result of all proteins involved in the TieDIE sub-network.

Hallmark main category	Hallmark sub-categories	*p*-value	Enrichment ratio	Gene number
Cell cycle	E2F Targets, G2M Checkpoint, p53 Pathway, Mitotic Spindle, Apoptosis	0.015443819	1.35328	29
DNA repair	PI3K/AKT/mTOR signaling, MTORC1 signaling	0.914616875	0.584921	7
Inflammatory response	IL2/STAT5 signaling, IL6/JAK/STAT3 signaling, inflammatory response, interferon alpha response, interferon gamma response, TNF alpha signaling via NFKB	0.010537424	1.56327	19
Metabolism	Hedgehog signaling, Myc targets, notch signaling, TGF beta signaling, WNT beta-catenin signaling	0.019642139	1.4941	18
Migration and invasion	Apical junction, epithelial/mesenchymal transition	5.84*E*−06	2.35103	24
Nuclear receptor signaling	DNA repair, UV response	0.199991976	1.16037	12
PI3K AKT mTOR	cholesterol homeostasis, fatty acid metabolism, glycolysis, reactive oxygen species pathway	0.029191187	1.58529	12
Stemness	Angiogenesis, hypoxia	0.302419603	1.06367	11
Tumor microenvironment	Androgen response, estrogen response early and late (merged to become nuclear receptor response)	0.401740261	0.966142	6

In addition, 10 cancer related hallmark pathways were also significantly enriched, including apoptosis pathway (*p*-value = 0.0221, cholesterol homeostasis pathway (*p*-value = 0.04152), epithelial/mesenchymal transition pathway (*p*-value = 0.0048), estrogen response early and late pathway (*p*-value = 0.04203), G2M checkpoint pathway (*p*-value = 0.0322), glycolysis pathway (*p*-value = 0.0048), Il6/Jak/Stat3 signaling pathway (*p*-value = 0.0067), mitotic spindle pathway (*p*-value = 0.0130), Mtorc1 signaling pathway (*p*-value = 0.0131) and TGF beta signaling pathway (*p*-value = 0.0500). Hallmark “wheels” were colored proportionally to the negative log transformed p-values returned by the hypergeometric test (Supplementary Table S7) (Figure 2D). These results further demonstrate the importance of our selected membrane proteins.

### Prognostic Value of Differentially Expressed Membrane Proteins in TieDIE Sub-Network (Overall Survival)

The OS prognostic value of the 32 membrane proteins in the TieDIE sub-network was evaluated by performing log-rank test in the TCGA lung cancer cohort (Supplementary Table S8). Based on the best cut-off of each gene, Kaplan–Meier (KM) curves were generated for the high expression level group and the low expression level group. The expression group definition is described in section “Survival Analysis.”

**FIGURE 3:**
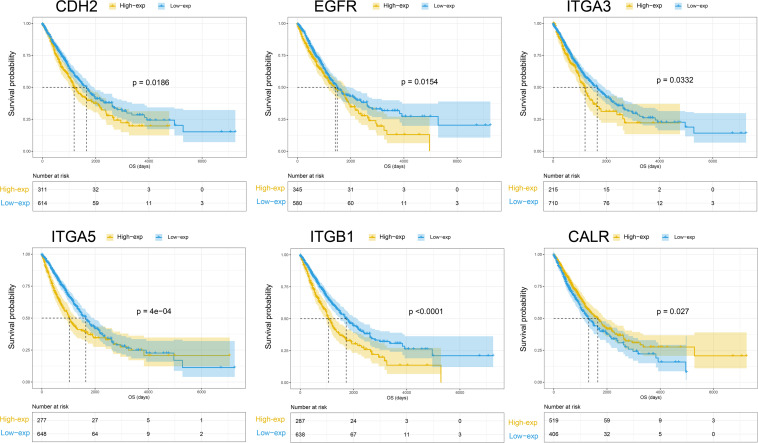
Kaplan-Meier curves of overall survival for CHE2, EGFR, ITGA3, ITGA5, ITGB1, and CALR.

High expression of CDH2 (HR = 1.2874, 95% CI: 1.0425–1.5898, *p* = 0.0279), EGFR (HR = 1.2927, 95% CI: 1.0496–1.5921, *p* = 0.0166), ITGA3 (HR = 1.2965, 95% CI: 1.0202–1.6477, *p* = 0.0379), ITGB1 (HR = 1.5930, 95% CI: 1.2906–1.9663, *p* = 2.1922e−05), and ITGA5 (HR = 1.4656, 95% CI: 1.1836–1.8148, *p* = 5.8933e−04) was negatively associated with OS in NSCLC patients. Low expression of CALR (HR = 0.7930, 95% CI: 0.6454–0.9744, *p* = 0.0279) was associated with worse OS for NSCLC patients (Figure 3). According to the differential expression analysis in this work; CDH2, EGFR, ITGA3, ITGA5, and ITGB1 were all highly expressed in the high- metastatic cell lines while CALR has low expression (see Supplementary Figure S1). This is consistent with the widely accepted fact regarding NSCLC – that patients with metastasis have a poor prognosis.

A Risk Index was computed for each sample by applying the formula described in the section “Materials and Methods.” When comparing the prognosis differences between the high and low risk factor groups, we found that the high risk group showed poor prognosis (*p*-value < 1e−05) (see Supplementary Figure S2). However, not all prognosis-associated genes match this pattern. We also found five other proteins – CD47, FN1, VDAC2, HGF, and ITGA4, which showed significant correlation with OS of NSCLC patients, however, the direction of the correlation was non-intuitive. HGF, ITGA4, and CD47 were also associated with increase in OS and we can observe that patients may live longer when these genes are highly expressed. However, high expression of HGF, ITGA4, and CD47 is observed in 95D cell lines (high metastasis cell lines) as compared with low metastasis cell lines. Finally, FN1 and VDAC2 are down regulated in the 95D cell lines but lower expression level of these proteins will result in longer OS (Supplementary Figure S1 – KM curve). A potential mechanistic explanation is due to inconsistent expression of protein level and gene level (Vogel and Marcotte, 2013).

### Validation of Altered Protein Expression

**FIGURE 4:**
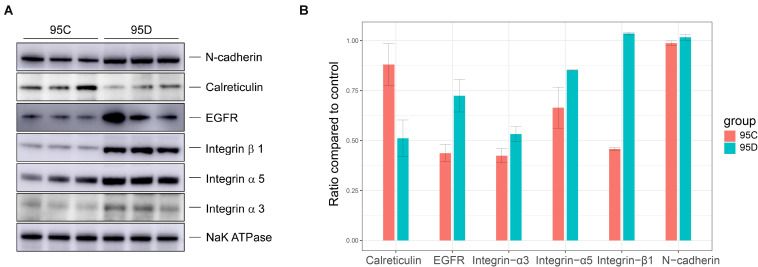
Western blot verification results. **(A)** Western blot assays of the protein level of CDH2, EGFR, ITGA3, TIGB1, TIGA5, and CALR. **(B)** The gray levels of Western blotting are shown by bar graph.

To validate the quantitative differences observed by mass spectrometry and bioinformatics analysis, the expression levels of six proteins were verified in the two (95C and 95D) cell lines through Western blot. The experimental results confirmed our prediction (Figure 4). Calreticulin (CALR gene production) showed low expression levels in high-metastasis cell lines (95D), while the other five proteins (CDH2, EGFR, ITGA3, ITGB1, and ITGA5) were highly expressed. The high expression of CDH2, EGFR, ITGA3, ITGA5, and ITGB1 and the low expression of CALR in lung cancer have been validated by experimental techniques (Western blot and/or immunohistochemistry) in previous studies (Boelens et al., 2007; Grinberg-rashi et al., 2009; Wang H. et al., 2013; Liu et al., 2015; Wu et al., 2016; Du et al., 2017). Combined with the results of this study, we can infer that from the onset of lung cancer, the high expression level of the five proteins (CDH2, EGFR, ITGA3, ITGB1, and ITGA5) and the low expression of ACALR may serve as biomarkers to determine whether the tumor has a high metastasis potential.

Besides biochemical validation of the six proteins (CDH2, EGFR, ITGA3, ITGB1, and ITGA5), we also performed literature verification. *N*-cadherin (CDH2), which is a member of the cadherin family and is involved in EMT and cancer metastasis (Hazan et al., 2004), has been reported as being highly expressed in LAC tissues. It was further revealed that LAC migration and invasion are suppressed after knocking down CDH2 (Zhao et al., 2013). Regarding epidermal growth factor receptor (EGFR), its gene amplification was shown as significantly increased in tumor cells and it was closely related to metastasis and TNM stage (Jia et al., 2015). Previous studies have verified the prognostic value of ITGA3, ITGA5, and ITGB1 expression on relapse and metastasis in lung cancer (Zheng et al., 2016). In addition, CALR was shown to be an independent prognostic factor for lung cancer (Liu et al., 2012), and the reduction in the expression of CALR was associated with an increased rate of proliferation (Bergner et al., 2009).

## Conclusion

The high metastatic status of giant cell lung cancer is strongly associated with the abnormal expression of membrane proteins, and microRNAs play a key role in regulation of expression. From this study, we conclude that the high expression of has-miR-137 and its indirect targets-CDH2, EGFT, ITGA3, ITGB1, ITGA5 and the low expression of CALR serve as markers of high-metastasis status of giant cell lung cancer. Our study provides a new approach to the analysis of integrated proteome and microRNAs and the synthesized sub-network provides candidate targets for giant-cell lung metastasis treatment.

## Data Availability Statement

The mass spectrometry proteomics data generated for this study was deposited into the ProteomeXchange Consortium via the PRIDE (Perez-Riverol et al., 2019) (https://www.ebi.ac.uk/pride/) partner repository with the dataset identifier http://www.ebi.ac.uk/pride/archive/projects/PXD016912.

## Author Contributions

YK participated in the data acquisition, performed the statistical analysis, and drafted the manuscript. ZQ and YR conceived of the study and participated in its design. GG contributed to the data interpretation and manuscript writing. MG conducted western-blot experiment and summarized results. All authors read and approved the final manuscript. All aspects of the study were supervised by HX, HL, and HZ.

## Conflict of Interest

The authors declare that the research was conducted in the absence of any commercial or financial relationships that could be construed as a potential conflict of interest.

## Footnotes

1 http://www.cbs.dtu.dk/services/TMHMM/

2 https://www.proteinatlas.org

## Supplementary Material

FIGURE S1 The KM curve of 5 genes on TCGA lung cancer cohort.

FIGURE S2 The KM curve of risk index on TCGA lung cancer cohort.

TABLE S1 Identification results of membrane proteins.

TABLE S2 Different expression analysis results between high and low metastatic cell lines.

TABLE S3 Validated microRNA-regulated targets network.

TABLE S4 GO and KEGG pathway the enrichment results.

TABLE S5 Gene set enrichment analysis results.

TABLE S6 Compact subnetwork predicted by TieDIE program.

TABLE S7 cancer hallmark pathway enrichment analysis of genes involved in subnetwork.

TABLE S8 Raw clinical data and 32 membrane protein expression data on TCGA lung cancer cohort.
