# Supplementary figures and images for: Integrative Analysis of Membrane Proteome and MicroRNA Reveals Novel Lung Cancer Metastasis Biomarkers
Source: Front Genet. 2020 Aug 28;11:1023. doi: 10.3389/fgene.2020.01023 (PMC7483668; doi:10.3389/fgene.2020.01023)

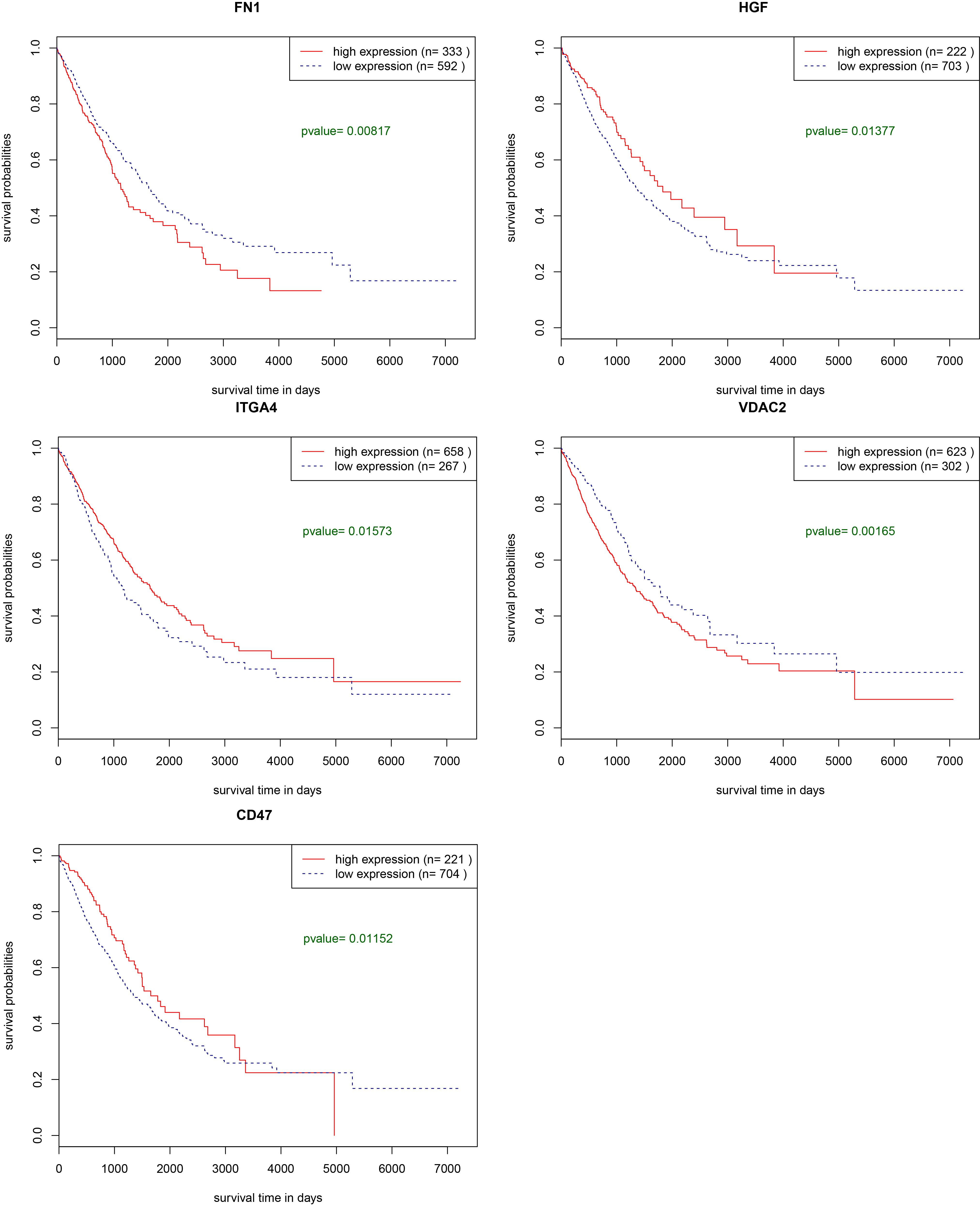

Supplement: FIGURE S1 — The KM curve of 5 genes on TCGA lung cancer cohort. [file Image_1.TIF]

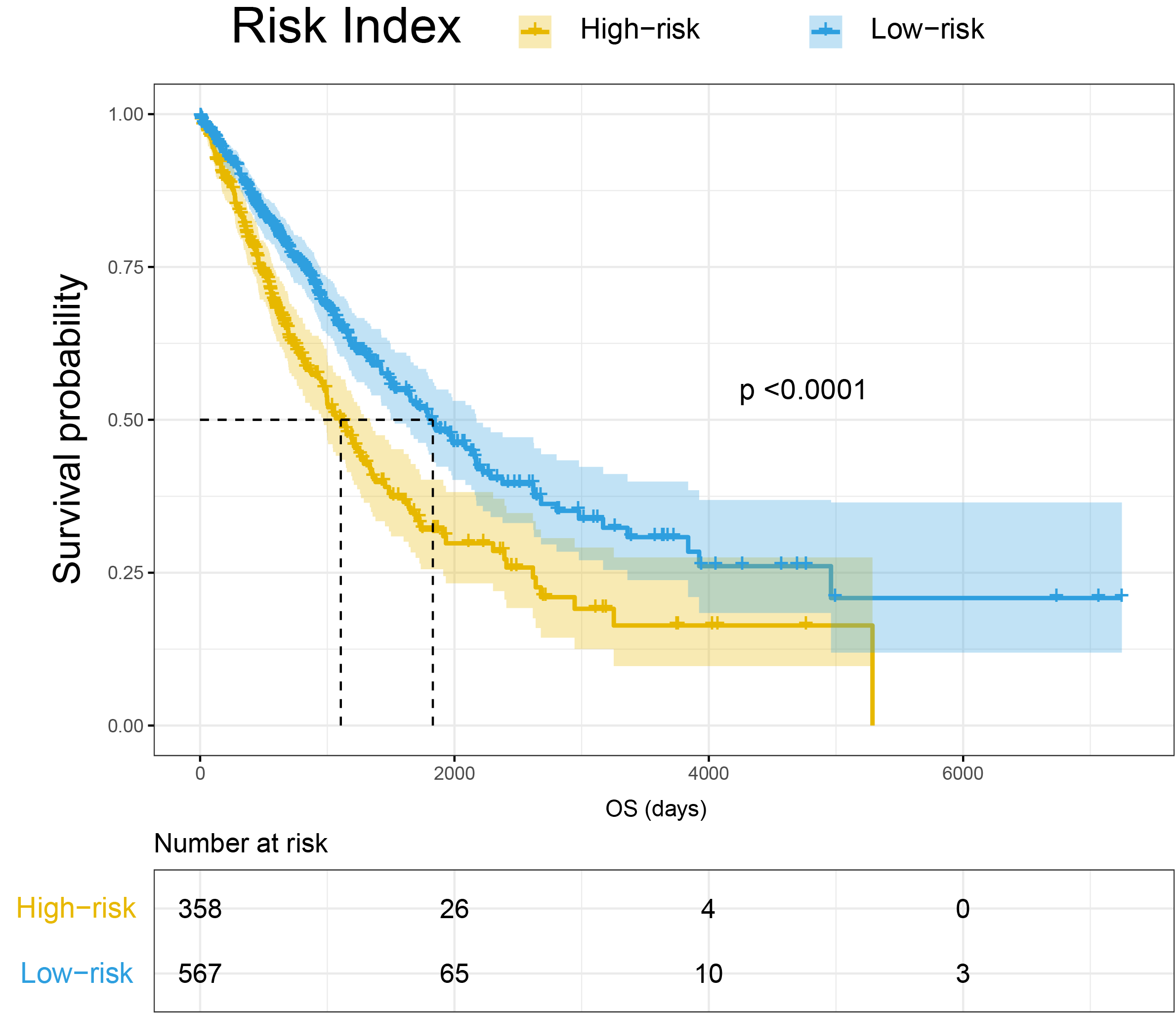

Supplement: FIGURE S2 — The KM curve of risk index on TCGA lung cancer cohort. [file Image_2.TIF]
